# Multifaceted mirror array illuminator for fluorescence excitation-scanning spectral imaging microscopy

**DOI:** 10.1117/1.JBO.28.2.026502

**Published:** 2023-02-07

**Authors:** Marina Parker, Samuel A. Mayes, Craig M. Browning, Joshua Deal, Samantha Gunn-Mayes, Naga S. Annamdevula, Thomas C. Rich, Silas J. Leavesley

**Affiliations:** a University of South Alabama, Department of Chemical and Biomolecular Engineering, Mobile, Alabama, United States; b University of South Alabama, Systems Engineering, Mobile, Alabama, United States; c University of South Alabama, Department of Pharmacology, Mobile, Alabama, United States; d University of South Alabama, Center for Lung Biology, Mobile, Alabama, United States

**Keywords:** hyperspectral imaging, fluorescence microscopy, excitation scanning, light emitting diode, spectroscopy, spectral imaging

## Abstract

**Significance:**

Hyperspectral imaging (HSI) technologies offer great potential in fluorescence microscopy for multiplexed imaging, autofluorescence removal, and analysis of autofluorescent molecules. However, there are also associated trade-offs when implementing HSI in fluorescence microscopy systems, such as decreased acquisition speed, resolution, or field-of-view due to the need to acquire spectral information in addition to spatial information. The vast majority of HSI fluorescence microscopy systems provide spectral discrimination by filtering or dispersing the fluorescence emission, which may result in loss of emitted fluorescence signal due to optical filters, dispersive optics, or supporting optics, such as slits and collimators. Technologies that scan the fluorescence excitation spectrum may offer an approach to mitigate some of these trade-offs by decreasing the complexity of the emission light path.

**Aim:**

We describe the development of an optical technique for hyperspectral imaging fluorescence excitation-scanning (HIFEX) on a microscope system.

**Approach:**

The approach is based on the design of an array of wavelength-dependent light emitting diodes (LEDs) and a unique beam combining system that uses a multifurcated mirror. The system was modeled and optimized using optical ray trace simulations, and a prototype was built and coupled to an inverted microscope platform. The prototype system was calibrated, and initial feasibility testing was performed by imaging multilabel slide preparations.

**Results:**

We present results from optical ray trace simulations, prototyping, calibration, and feasibility testing of the system. Results indicate that the system can discriminate between at least six fluorescent labels and autofluorescence and that the approach can provide decreased wavelength switching times, in comparison with mechanically tuned filters.

**Conclusions:**

We anticipate that LED-based HIFEX microscopy may provide improved performance for time-dependent and photosensitive assays.

## 1 Introduction

**Fig. 1:**
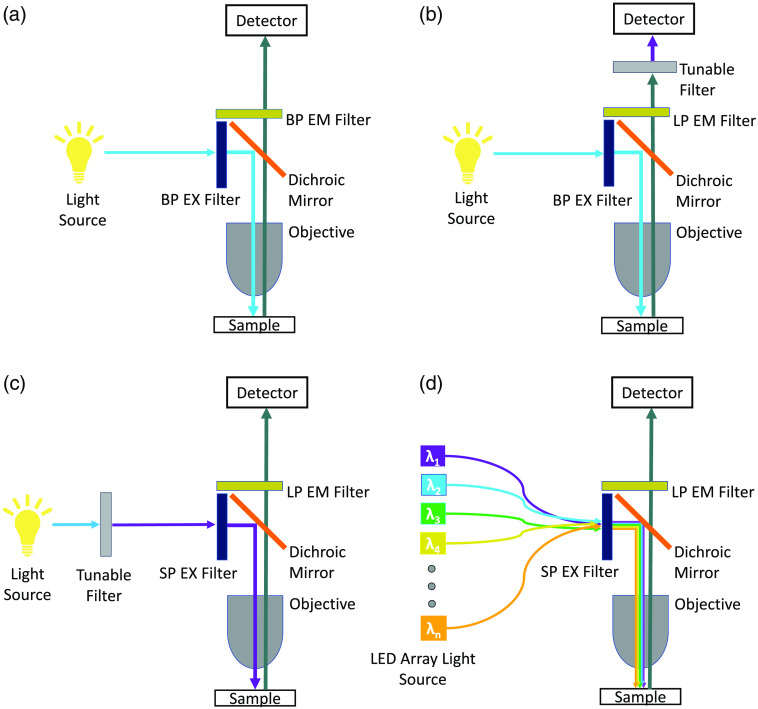
(a) Simplified light path for a standard epifluorescence microscope. Light emitted from a broadband light source passes through a band-pass excitation (BP EX) filter and is reflected by a dichroic mirror to illuminate the sample. Fluorescence emission from the sample passes through the objective and the dichroic beam splitter and is filtered using a band-pass emission (BP EM) filter and is detected. (b) A typical emission scanning HSI light path implemented in an epifluorescence microscope. Light emitted from the light source is filtered through a BP EX filter and is reflected by a dichroic mirror to illuminate the sample. The fluorescence emission passes through the dichroic mirror and a long-pass emission (LP EM) filter and is then spectrally filtered and detected. This technique results in additional light loss due to the tunable filter or other spectral element and may require increased acquisition times. (c) An excitation-scanning HSI light path based on a tunable filter implemented in an epifluorescence microscope. Excitation light emitted from the light source is filtered through a tunable filter, passed through a short-pass excitation filter, and reflected by a dichroic mirror to illuminate the sample. The fluorescence emission passes through the dichroic mirror and long-pass emission filter, and the emission above a characteristic cutoff wavelength is detected. (d) LED-based HIFEX scanning light path implemented on an epifluorescence microscope. In this approach, a series of wavelength-specific LEDs provides spectral excitation, which passes through a short-pass excitation filter and is reflected by a dichroic mirror to illuminate the sample. The fluorescence emission passes through the dichroic mirror and long-pass emission filter, and the emission above a characteristic cutoff wavelength is detected.

Traditional fluorescence microscopy utilizes a broadband light source and a series of optical band-pass and dichroic filters to illuminate a fluorophore at a peak excitation wavelength (λ) and detect emission at a peak emission wavelength [Fig. 1(a)]. This approach works well for detecting fluorescence from a single label or several spectrally well-separated labels by utilizing several band-pass filters to isolate the excitation and emission wavelengths of respective fluorescent labels.^1^ However, many assays require additional methods to separate signal crosstalk between labels, and traditional fluorescence imaging approaches may not adequately allow this signal separation, resulting in a lack of specificity and potentially creating image artifacts. Subtraction of signal crosstalk between probes can be further complicated when labels share similar excitation or emission peak wavelengths.^1^ In addition, the emission spectrum of the tissue autofluorescence often covers a wide spectral range that overlaps the emission of many fluorescent probes, resulting in difficulty separating signals from labels and autofluorescence.^2^^–^^5^

Emission scanning hyperspectral imaging (HSI) [Fig. 1(b)] overcomes limitations of standard fluorescence imaging through the ability to sample image data at many wavelength bands, forming a contiguous spectrum.^1^^,^^6^^–^^9^ Applied to fluorescence microscopy, this technique excites the sample at one or several excitation peak wavelengths and collects a range of emission wavelengths of narrow bandwidth, allowing the user to analyze fluorescence emission from multiple labels through linear unmixing or other spectral analysis approaches.^6^^,^^10^^–^^14^ However, spectral filtering technologies can present a major constraint of this approach as they require the use of tunable filters^8^^,^^13^^,^^15^ or dispersive mechanisms, such as prisms^15^^–^^17^ or gratings,^9^^,^^15^ that often attenuate the emitted light, reducing the signal available for detection and thus reducing the signal-to-noise characteristics of the detected image. Due to light losses associated with spectral filtering, emission-scanning HSI techniques have typically been slow and prone to photobleaching.

Excitation scanning HSI can provide improvements in photon efficiency when compared with similarly configured emission scanning systems. In excitation scanning, the excitation light is filtered and used as the basis for spectral discrimination, rather than the emission, thus allowing all emitted light^1^ above a characteristic cutoff wavelength to be detected [Fig. 1(c)]. We previously demonstrated the effectiveness of this approach, which we coined hyperspectral imaging fluorescence excitation scanning (HIFEX), using a xenon arc lamp as a light source and a series of thin film tunable filters (TFTFs) to provide a tunable excitation band.^5^^,^^18^^–^^20^ The TFTF array enabled the selection of a range of narrow-band excitation wavelengths. Our initial HIFEX prototype demonstrated improved acquisition speeds and signal-to-noise ratios (SNRs) in a side-by-side comparison with emission scanning. However, the system was limited by the time required to mechanically shift between excitation wavelengths—wavelength switching required up to 250 ms. Despite the slow wavelength switch times, our results showed that a >10× higher signal could be detected using HIFEX due to it detecting a broad band of emitted light. Moreover, excitation scanning provided improved delineation of nuclear and cell borders and increased identification of fluorescently labeled regions in highly autofluorescent tissue when compared with similarly configured emission scanning.^1^^,^^12^^,^^13^

Here, we describe an alternative approach for HIFEX that utilizes an array of LEDs as the spectral excitation source [Fig. 1(d)]. Compared with the previous TFTF-based approach, the LED-based HIFEX system provides greatly improved wavelength switching speeds. An optical geometry was developed^21^^,^^22^ to allow for up to 16 predetermined excitation wavelengths while providing output through a standard liquid-light guide that was coupled to the microscope. To our knowledge, this is the first time that a multifaceted mirror geometry has been used to combine output from up to 16 single-band LEDs for spectral illumination. This approach provides a greater number of wavelength bands than current commercially available LED-based microscope illumination sources (Table 1).

**Table 1:** Comparison of LED-based HIFEX with current commercial systems or hyperspectral modules.

Emission-scanning hyperspectral imaging systems						
${\text{Description}}^{\text{references}}$	λ range (nm)	Max. # of λ/spectral image	Acquisition speed (fps)	Spatial sampling (MP)	Cost	Sensitivity (% emitted signal/channel)
Spectral laser scanning confocal microscope^10^^,^^23^^,^^24^ (Nikon A1R, Zeiss 980, etc.)	400 to 750 (adjustable)	32 to 34	0.1	∼4	$$$	∼0.5%
Spinning disk^25^^,^^26^ confocal microscope (Andor)	405 to 640 (adjustable)	4	1 color: 1600	∼4	$$$	∼10%
			2+ colors:1			
Spectral laser resonance scanning^27^^,^^28^	450 to 1100, 680 to 1300, or 800 to 1800	4	45	∼1	$$$	∼5%
Excitation-scanning HSI technologies						
${\text{Description}}^{\text{references}}$	λ range (nm)	Max. # of λ/spectral image	Acquisition speed (spectral images/min)	Spatial sampling (MP)	Cost	Sensitivity (% emitted signal/channel)
Xe Arc Lamp + AOTF^8^^,^^13^^,^^29^	350 to 650 or 400 to 750	Tunable (∞)	∼1 to 20 (photon limited)	∼1 to 9 (camera specific)	$$	∼95%
Xe Arc Lamp + TFTF^1^^,^^8^^,^^15^^,^^30^	360 to 650	Tunable (∞)	∼1 to 10 (mechanical tuning limited)	∼1 to 9 (camera specific)	$$	∼95%
Commercial multi-LED^31^	365 to 750 (discrete values)	4 to 6 (some systems feature 16 channels to select between, only four available for a given spectral image)	∼120 or 240	∼1 to 9 (camera specific)	$	∼95%
Proposed/prototype system	365 to 560	Up to 16 (11 demonstrated in current prototype)	∼10 to 20 (currently ex. photon limited)	∼1 to 9 (camera specific)	$	∼95%

## 2 System Materials, Methods, and Validation

The system concept and design were based on the goal of developing innovative approaches to improve acquisition speeds in hyperspectral microscopy using fluorescence excitation scanning. To address the wavelength switching speed imitations of our previous TFTF-based HIFEX system, wavelength-specific LEDs, which provide on/off times of ∼10 to 20  μs, were implemented. The overall system design process includes the following: (1) conceptual designs were drawn using Inventor (Autodesk, Inc., San Rafael, California); (2) parametric modeling and *in silico* optimization was performed using TracePro (Lambda Research Corp.), a Monte Carlo-based optical ray trace package; (3) optimized optical geometries were prototyped; (4) benchtop testing and *in situ* optimization were performed using optical power meter and other test equipment; and (5) feasibility testing was performed by coupling to an inverted microscope platform.

### 2.1 Conceptual Design

**Fig. 2:**
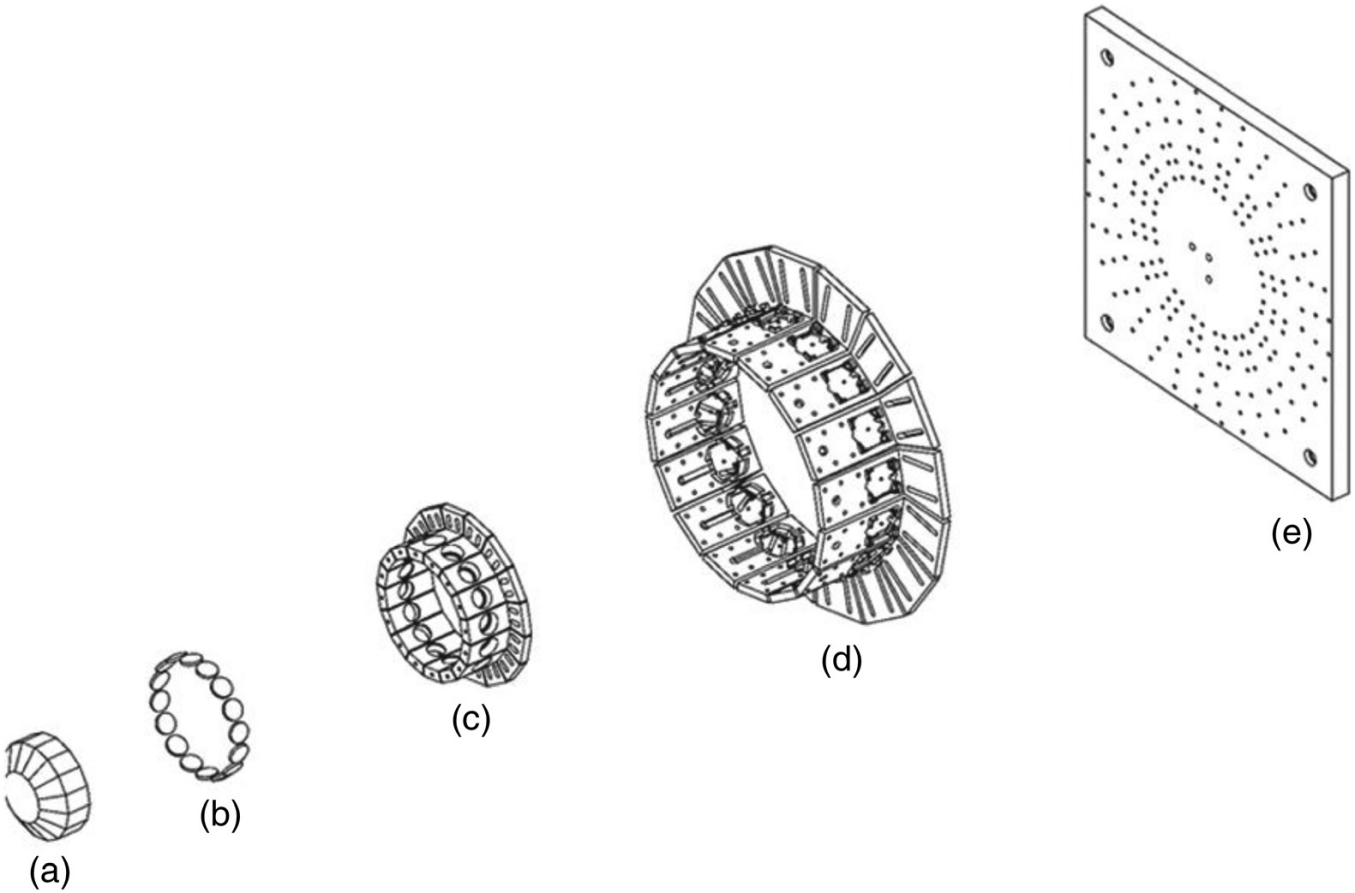
Exploded assembly view of the main components of the LED-based spectral illumination system. (a) The multifaceted mirror array, (b) lenses, (c) lens holders, and (d) LED in LED holders were mounted on (e) a custom baseplate.

**Fig. 3:**
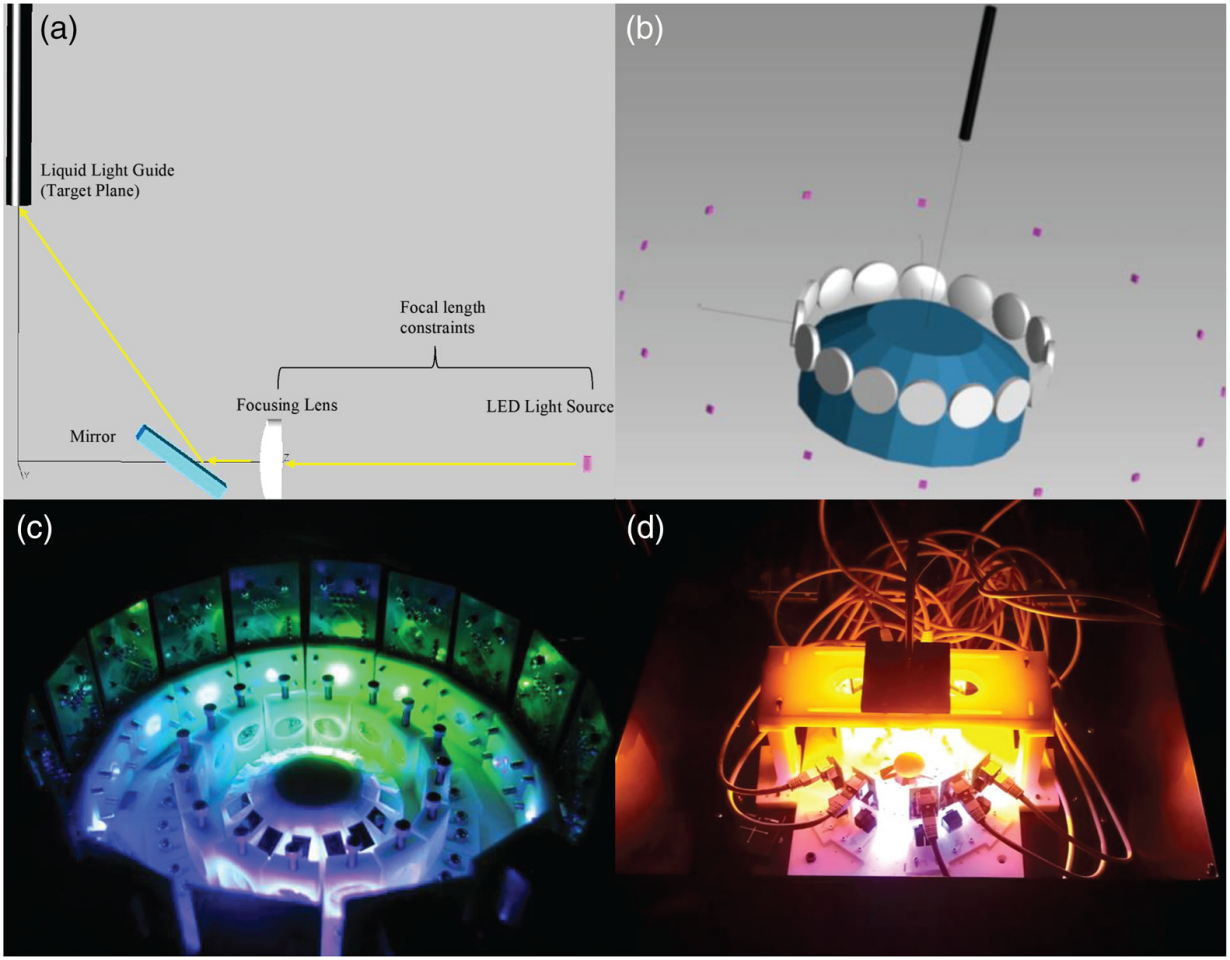
Overview of the excitation scanning HSI LED system design. (a) A simplified sketch showing the light path (yellow) from the LED (pink) through a focusing lens (white), and reflected by a mirror (blue) and onto the entrance aperture of the LLG (black). (b) A visualization of the optical components as simulated in the TracePro optical ray trace model environment. LEDs (pink), lenses (gray), the multifaceted mirror array (blue), and the LLG (black) are shown. (c) Photograph of an early prototype system utilizing individual mirrors instead of the multifaceted mirror. For visualization purposes, all LEDs were illuminated simultaneously. (d) Photograph of a revised prototype system utilizing the multifaceted mirror array and connected to the LLG, as seen by the black tube exiting the top of the illumination module.

The optical design utilizes a custom machined 16-faced multifaceted mirror array to reflect LED illumination onto the entrance aperture of a liquid light guide (LLG), which delivers light to the microscope. Optical alignment components were designed using Inventor software and include lens holders, LED holders, and parts used to assemble components of the system (Fig. 2); an example light path is shown in Fig. 3(b). Light is emitted from each LED [Fig. 2(d)] one at a time and then collimated by a lens [Fig. 2(b)] mounted in a lens holder [Fig. 2(c)] that can be positioned to collimate the light. The light is then reflected upward by a multifaceted mirror array [Fig. 2(a)] and is incident at a common focal focus location, which corresponds to the entrance aperture of the LLG.

### 2.2 Lens and Liquid Light Guide Properties

Lens files were obtained from the manufacturer (Edmund Optics) in Zemax format and imported into TracePro. Lenses with the following focal lengths (FLs) were evaluated: 19.1, 38.1, and 31.8 mm. For the final prototype, the 31.8-mm FL lens was selected. All lenses were 12.7 mm in diameter and plano-convex ${\mathrm{MgF}}_{2}$ coated.

A 3-mm-diameter active core, Lumatec series 300 (Sutter Instruments) LLG was used for coupling to an inverted microscope. Properties of the LLG are listed in Table 2.

**Table 2:** Lumatec liquid lightguide property table.

Series	Core diameter (mm)	NA 2α	Spectra ranges (nm)	Specific properties
300	3	72 deg	280 to 650	–Transmission of up to 5W of UV radiation
				–Suitable for rugged environments
				–Compatible light sources: mercury and Zenon short arc, tungsten halogen, LED
				–Temperature range (long term): −5°C to +35°C

The LLG was modeled as a cylinder with a 3 mm diameter and 1 mm thickness. The target plane (interrogation plane) was defined as the entrance aperture of the LLG.

### 2.3 Multifaceted Mirror Array

The multifaceted mirror array was designed in Inventor and imported into TracePro, and optical characteristics were defined as a “perfect reflector.” The mirror array angle was preselected to achieve a beam angle of 22.5 deg (half angle) at the entrance of the LLG, so light that was incident on the LLG entrance aperture would fall within the acceptance angle of the LLG. In addition, Inventor files were sent to Space Optic Research Labs (Merrimack, New Hampshire) for prototyping. The mirror was machined from aluminum with antireflection coating.

### 2.4 Parametric Modeling

Theoretical parametric modeling was performed for a range of optical and geometric variables, including different FL lenses and geometric location of components. To analyze the theoretical feasibility of our approach, a ray trace model that evaluated light transmission from 11 LEDs was developed. The ray trace model accounted for the LED output, focusing lenses, the multifaceted mirror array (Table 3), and the LLG entrance aperture diameter. LED properties were defined in TracePro, and lens and mirror array properties were imported from manufacturer-supplied data files. LEDs were modeled as a 1  mm×1  mm×1  mm cube with one surface assigned at the emitter surface. A range of different FL lenses (Table 3) were also modeled. To account for wavelength-dependent attenuation and further optimize the optical power available at the entrance aperture of the LLG, the TracePro macrolanguage was utilized to perform a parametric sensitivity study that evaluated a range of geometric locations of the LEDs and the final selected prototype lens. The LLG was modeled in TracePro as a cylinder with a 3-mm entrance aperture and placed at a fixed z-axis position of 63 mm above the mirror array, which is governed by the acceptance angle of the reflected light rays. Properties of the LLG were not included in the model because only the entrance aperture of the LLG was used to assess the total transmission of light from each LED. Ray trace modeling results were evaluated by measuring the optical power available and the angular power distribution at the entrance aperture of the LLG for each LED wavelength illuminated. The transmission efficiency was calculated as the ratio of optical power incident on the LLG entrance aperture to total power output of the LED.

**Table 3:** Optical and optoelectronic components used for parametric modeling.

LED	Parameters	Provider
SMB1N-365V-02	Dimension: 5.0 × 5.2 × 5.5 mm, viewing angle: 18 deg	Roithner LaserTechnik GmbH
SMB1N-375V-02		
SMB1N-395V-02	Dimension: 5.0 × 5.2 × 5.5 mm, viewing angle: 22 deg	
SMB1N-405V-02	Dimension: 5.0 × 5.2 × 5.5 mm, viewing angle: 20 deg	
SMB1N-420H-02	Dimension: 5.0 × 5.2 × 5.5 mm, viewing angle: 22 deg	
SMB1N-D450-02		
SMB1N-D470-02	Dimension: 5.0 × 5.2 × 5.5 mm, viewing angle: 20 deg	
SMB1N-490H-02	Dimension: 5.0 × 5.2 × 5.5 mm, viewing angle: 22 deg	
SMB1N-515V-02	Dimension: 5.0 × 5.2 × 5.5 mm, viewing angle: 18 deg	
SMB1N-D520V-02	Dimension: 5.0 × 5.2 × 5.5 mm, viewing angle: 20 deg	
SMB1N-525V-02	Dimension: 5.0 × 5.2 × 5.5 mm, viewing angle: 22 deg	
Lens	Parameters	Provider
49586 – ${\mathrm{MgF}}_{2}$ coated, plano convex	12.7 mm diameter × 19.1 mm FL	Edmund Optics
49858 – ${\mathrm{MgF}}_{2}$ coated, plano convex	12.7 mm diameter × 31.8 mm FL	
49859 – ${\mathrm{MgF}}_{2}$ coated, plano convex	12.7 mm diameter × 38.1 mm FL	
**LLG**	**Parameters**	**Provider**
Liquid light guide	3 mm core diameter, NA 2α=72°	Sutter
**Multifaceted mirror**	**Parameters**	**Provider**
Custom mirror	—	Space Optics Research Lab

#### 2.4.1 Validation of LED source properties

**Fig. 4:**
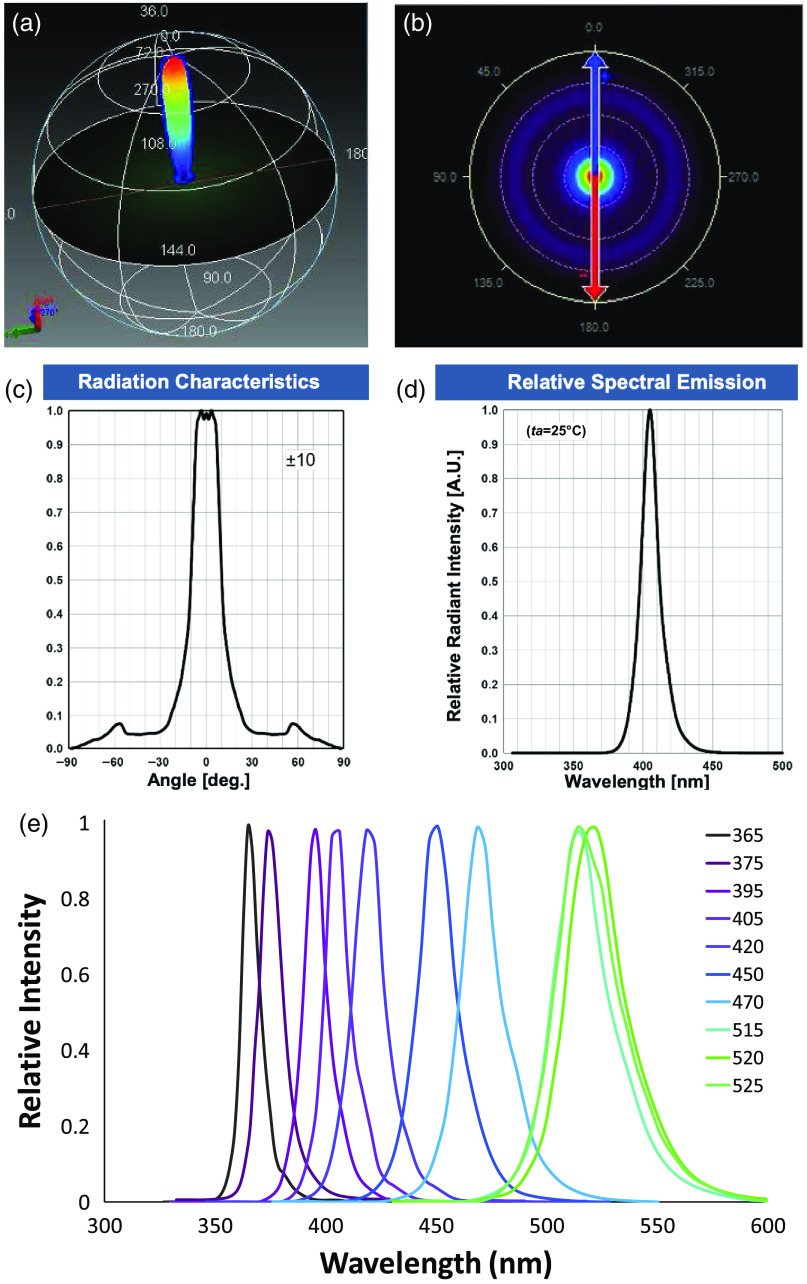
(a) TracePro source property tool was used to extract the 3D beam profile, (b) polar angular distribution, (c) radiation characteristics, and (d) relative spectral emission of each of the LEDs (405-nm LED shown for example). (e) A range of LED spectral profiles were evaluated.

LED source properties were extracted from manufacture data sheets to build an optical component library using the source property tool in TracePro (Fig. 4). LED optical properties were experimentally tested to validate manufacturer specifications (data not shown). For each LED, a surface source property was created in Trace Pro that accounted for the angular divergence and distribution, optical intensity, and spectral bandwidth characterized by manufacturer data. Surface properties were assigned to the designated emitter surface of each LED.

### 2.5 System Prototyping

A prototype was constructed to evaluate the feasibility of the optimized model geometry for spectral imaging microscopy.

#### 2.5.1 Optomechanical prototyping

A multifaceted mirror array was designed in Autodesk Inventor and fabricated by Space Optics Research Labs, LLC. The array contained 16 mirror facets of 10  mm×12  mm aligned at evenly distributed angles across 360 deg in the horizontal (XY) plane and specified at a 55 deg angle with respect to the vertical (Z) plane. The vertical angle was selected to reflect a horizontal beam from each LED to the entrance aperture of the LLG while achieving a half angle of 22.5 deg with respect to the angular acceptance of the LLG. Optical alignment parts were designed using Inventor, processed for three-dimensional (3D) printing using Simplify 3D, and printed using a MakerGear M2 Rev.E. dual extruder 3D printer. Optomechanical alignment parts consisted of an LED circuit board mount, a lens mount, and a custom optical breadboard. All housing parts were printed used acrylonitrile butadiene styrene filament.

#### 2.5.2 Electronic prototyping

Electronic hardware consisted of a relay board (RLY102-24V, Winford Engineering), DC power supply unit (VF-S250-24A-CF, CUI, Inc.), four digital/analog breakout boards (CB-68LPR, National Instruments), analog computer interface board (PCI-6723, National Instruments), and digital computer interface board (PCIe-6323, National Instruments). Custom electronic interface boards were designed for routing analog and digital control signals from National Instruments cards to respective LED current drivers. The control board was used to split analog and digital lines into 16 ethernet ports. Ethernet cables were used to connect the control board to each LED circuit. An analog reference voltage was used to adjust a variable current driver (945-1132-ND, RECOM), which regulated the current to each LED, and a digital TTL signal was used to switch the driver on or off.

### 2.6 Benchtop Testing

#### 2.6.1 LED spectroradiometric testing

Optical power measurements were performed using a fiber-coupled spectrometer (QE65000, Ocean Optics, Inc.) connected to an integrating sphere (FOIS-1, Ocean Optics, Inc.) and calibrated using an NIST-traceable lamp (LS-1-CAL-INT, Ocean Optics, Inc.). The power output versus reference voltage ($V_{\text{ref}}$) curve for each LED was characterized and used to optimize the optical position and alignment of LEDs and lenses. Optical power measurements of the overall system, as connected to an inverted fluorescence microscope, were also made by placing the integrating sphere on the sample stage immediately above the objective and measuring the intensity of each LED. Measurements taken at the stage represent the total illumination for each channel delivered to the target, allowing for correction of subsequent spectral images to a flat spectral response.

#### 2.6.2 Imaging software setup

NIS Elements (Nikon Instruments, Inc.) software was used to control the spectral light source, microscope, and camera. The “triggered acquisition” panel was used to control the analog and digital signals and camera operation to coordinate the excitation wavelength, intensity, and image acquisition. Wavelength switching was externally triggered using the camera shutter signal.

### 2.7 Spectral Imaging Feasibility Testing

Validation and feasibility testing were performed by comparing imaging results of the custom LED-based HIFEX spectral microscope system with those obtained using the prior HIFEX system that utilized a TFTF array for tunable excitation. Results were also compared with a commercially available Zeiss LSM 980 (Carl Zeiss Microscopy, LLC, White Plains, New York).

#### 2.7.1 Cell sample preparation

Custom six-label slides consisting of African green monkey kidney epithelial cells were purchased from Abberior GmbH. Labels were selected to span a wide spectral range to allow for the evaluation of the spectral separation capabilities (Table 4). In addition to the six-label slide, single-label control slides, as well as an unlabeled control to assess autofluorescence contributions, were also purchased. Cells were fixed and stained by Abberior in accordance with Wurm et al.,^32^ and all cell specimens were embedded in Abberior Mount Solid Antifade (item number: MM-2013-2X15ML).

**Table 4:** Slide number and staining conditions for each slide used in the six-label imaging experiment. Excitation and emission peak wavelengths shown are those reported by the manufacturer, with the exception of cellular autofluorescence, for which peak wavelengths were estimated from the experimental data recorded in this study.

Slide #	Stain	Ex. peak λ (nm)	Em. peak λ (nm)
1	Fixed and unstained cell slide (autofluorescence)	480	505
2	F-actin: Abberior STAR GREEN phalloidin	493	519
3	Mitochondria: Abberior STAR520SXP goat anti-rabbit IgG	515	612
4	Double-stranded DNA: Abberior LIVE 560 DNA	561	584
5	Vimentin: Abberior STAR ORANGE goat anti-chicken IgY	589	616
6	Golgi apparatus: Abberior STAR RED goat anti-guinea pig IgG	638	655
7&8	Multilabeled slides with labels of slides 1 to 6	Mixed	Mixed

#### 2.7.2 Microscope integration

The spectral light source was coupled via LLG to an inverted epifluorescence widefield microscope (TE2000-U, Nikon Instruments, Melville, New York), equipped with a 60×-water immersion objective (Plan Apo VC 60x/1.2 WI, Nikon Instruments). Images were acquired using a Prime 95B sCMOS camera (Teledyne Photometrics, Tucson, Arizona).

#### 2.7.3 Image acquisition

Images were acquired for each sample (six-label slide, single-label control slides, and unlabeled autofluorescence control slide) on each of three spectral microscope systems (Zeiss LSM 980, HIFEX with TFTF array, and new HIFEX with LED excitation). Imaging parameters were set accordingly based on the system used (Table 5).^33^

**Table 5:** Summary of microscope configuration and image acquisition parameters.

Microscope	Light source	Objective lens parameter(s)	Detector
Inverted epifluorescence widefield microscope (TE2000-U, Nikon Instruments, Melville, New York)	TFTF array	Plan Apo VC 60×/1.2 WI (Nikon Instruments)	Prime 95B sCMOS camera (Teledyne Photometrics, Tucson, Arizona)
	Scan range of 360 to 545 nm		
	5 nm increments		250 ms per wavelength band
			4 averaging
	Power: 100%		2×2 binning
			0 gain, 0 offset
			40 MHz readout speed
	LED-based HIFEX		Prime 95B sCMOS camera (Teledyne Photometrics, Tucson, Arizona)
	LED wavelengths (nm): 365, 375, 385, 405, 415, 420, 430, 450, 470, 490, 515, 520		
			1s per wavelength band
			4 averaging
			2×2 binning
			0 gain, 0 offset
	Power: 100%		40 MHz readout speed
Zeiss LSM 980 commercial emission-scanning spectral confocal microscope system (Carl Zeiss Microscopy, LLC, White Plains, New York)	Laser	C-Apochromat 40×/1.2W autocorrect	32 channel GaAsP-PMT
	405 nm at 10% power		4 averaging
			2 min 22 s exposure/dwell time
	488 nm at 2% power		
	561 nm at 0.2% power	UV VIS IR (Zeiss Microscopy)	0 offset, 0 binning
	639 nm at 2% power		Digital gain 1
			Gain 650 V

#### 2.7.4 Image analysis

For both HIFEX systems, wavelength-dependent excitation intensity was corrected to a flat spectral response as described previously.^3^ In brief, a spectral correction coefficient was calculated for each wavelength band to account for the wavelength-dependent illumination intensity. For the TFTF-based system, this was performed by tuning the system to each wavelength band in the spectral image scan range and measuring the optical power output at the microscope stage using a fiber-coupled spectrometer and integrating sphere. For the LED-based system, this was performed by sequentially illuminating each LED (at maximum power output) and measuring the optical power output available at the microscope stage. Hence, separate correction factors were calculated and used for the TFTF- or LED-based system. Spectral image stacks were then corrected by first identifying a blank (background) region in each image and extracting the mean spectrum from that region. The mean background spectrum was then subtracted from each spectral image stack, and the stack was subsequently multiplied by the spectral correction factor to correct the image to a flat spectral response [Eq. (1)]:

$$I_{\text{corrected}}=(I_{\text{raw}}-I_{\text{dark}}\cdot \mathrm{CC}),$$ (1)

where $I_{\text{raw}}$ is the original spectral image, $I_{\text{dark}}$ is the background spectrum, and CC is the spectral correction coefficient. Spectral image data from the LSM 980 were not corrected as the spectral detector is factory calibrated to provide a flat spectral response. All corrections were performed using a custom MATLAB script (MathWorks, Inc., Natick, Massachusetts).

Corrected spectral images were then processed to build a spectral library from single-label control samples, as described previously.^1^^,^^3^^,^^5^ In brief, regions of high signal strength were selected within spectral images from each single-label control sample, as well as the unlabeled autofluorescence control. The mean spectrum from each region was extracted and saved within a library for spectral unmixing. Due to high cellular autofluorescence, for a subset of samples, a fraction of the autofluorescence spectrum was subtracted from the single-label spectrum before saving the single-label spectrum within the spectral library. The test image of the six-label sample was then linearly unmixed using a non-negatively constrained linear unmixing algorithm, which provides a quantitative estimate^5^^,^^11^^,^^12^ for the abundance of each label (also known as an endmember) within each pixel. Unmixed images were linearly scaled, false colored, and overlayed for visualization.

An example spectral unmixing code is available in Ref. 34.

#### 2.7.5 SNR measurements

To quantify performances of each system, SNRs were estimated in each unmixed gray scale image stack using an approach described by Amer et al.^35^ for estimation of noise in video images that was previously adapted for fluorescence microscopy by Bernas et al.^36^ and utilized in Refs. 1 and 3. In summary, each image was analyzed by utilizing an eight-way (eight-directional) high-pass filter to identify pixels within regions of homogeneous intensities. Surrounding pixels were then quantified to determine the estimated standard deviation associated with each pixel. The SNR was obtained by dividing the mean intensity of each pixel by the standard deviation of each pixel, and a median SNR of at least five regions was used as the SNR for each image.

## 3 Results and Discussion

We present results for revised excitation-scanning spectral imaging technology based on illumination with many narrow-band LEDs that overcomes the wavelength switching-speed limitations of previous approaches. An optical approach is utilized to combine the wavelength output from the narrow-band LEDs using a multifurcated mirror. The following sections present the parametric modeling, prototyping, benchtop testing, and feasibility testing results from this system.

### 3.1 Parametric Modeling Results and Discussion

**Fig. 5:**
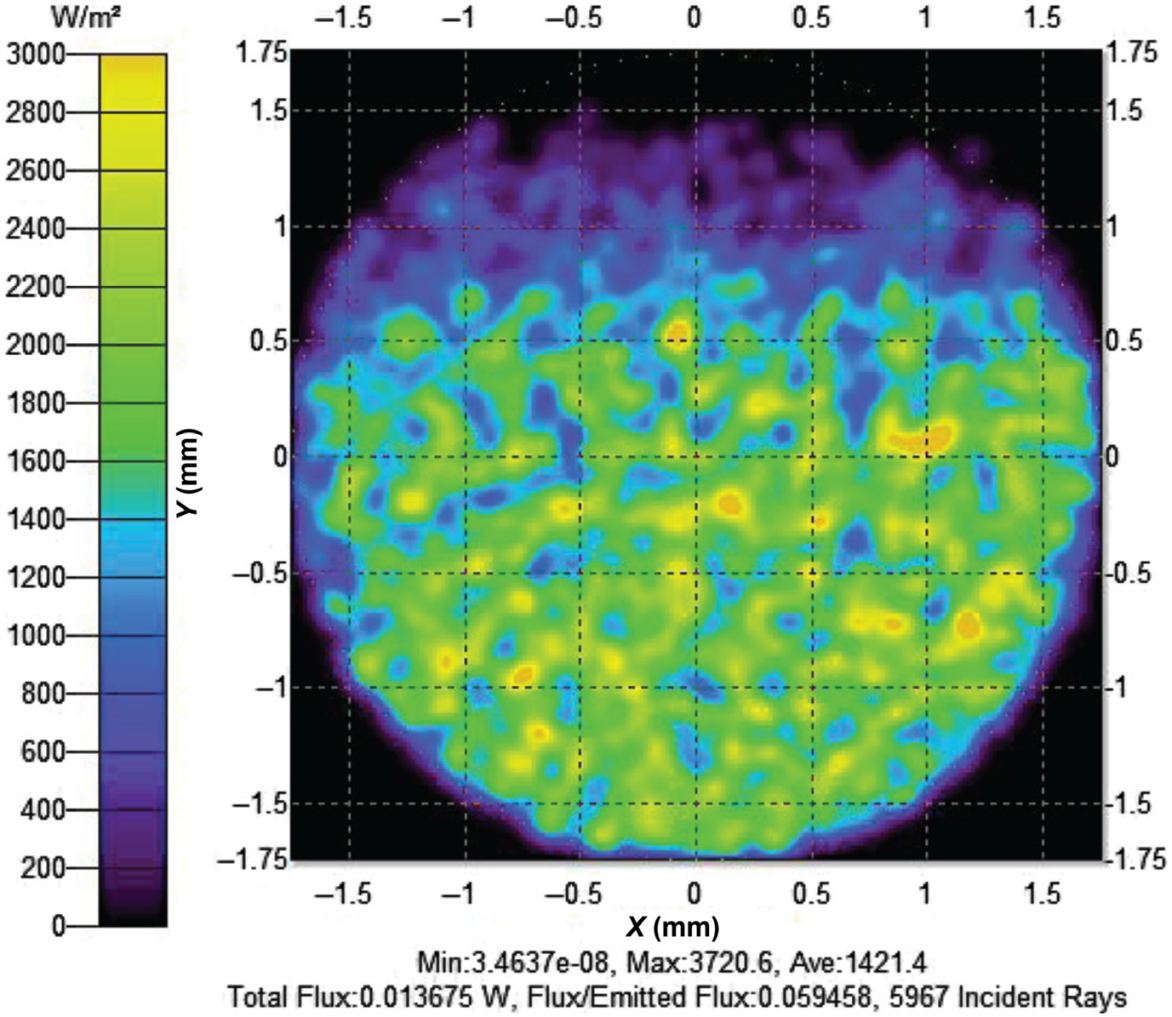
Example of the irradiance map used to evaluate transmission data and the spot size at the entrance aperture of the LLG, which is located at z=63  mm above the mirror array. The map shown here is for the 525-nm LED placed at $x_{\mathrm{LED}}=84\;\mathrm{mm}$ and FL lens of 31.08 placed at $x_{\text{lens}}=37\;\mathrm{mm}$ away from the mirror array. The irradiance map is a representation of the illumination intensity distributed over a measurement surface. It is achieved by visualizing the distribution of all rays divided by total initial emitted power of the LED. For the 525-nm LED, total flux/emitted flux is 0.059458 or ∼5.95%.

Parametric modeling, using TracePro ray trace software, was used to evaluate lens options and optimize the location and orientation of components. Sensitivity studies, using a range of parameter values, were run using the TracePro macro language. Irradiance maps (Fig. 5) were used to evaluate transmission data and the spot size at the LLG. An irradiance map is the irradiance in watts per unit area or lux that is incident or absorbed on the selected surface, which in the parametric model is the entrance aperture of the LLG. Results from the lens sensitivity study considered three different FL lenses (19.1, 38.1, and 31.8 mm) and included the optical power available at the entrance aperture of the LLG, the corresponding percent transmission of the system, the angular power distribution, and a visualization of the spatial distribution of power (irradiance map) on the entrance aperture of the LLG, which was saved as an image file. Results showed that the 31.8-mm FL lens provided the highest percent flux (5.946%) at the entrance aperture of the LLG when the lens and LED were placed at $x_{\text{lens}}=37\;\mathrm{mm}$ and $x_{\mathrm{LED}}=84\;\mathrm{mm}$ away from the mirror array, respectively (Fig. 5). The 19.1-mm FL and 38.1-mm FL lenses provided a maximum flux percentage of 5.409% and 3.926% with lens and LED placements at $x_{\text{lens}}=30\;\mathrm{mm}$ and $x_{\mathrm{LED}}=56.1\;\mathrm{mm}$ and $x_{\text{lens}}=41\;\mathrm{mm}$ and $x_{\mathrm{LED}}=100.1\;\mathrm{mm}$ away from the mirror array, respectively. Therefore, lens 49858 (${\mathrm{MgF}}_{2}$ coated, olano convex, 12.7 mm diameter × 31.8 mm FL) was chosen as the prototype lens. Because the lens sensitivity studies were only performed using the 525-nm LED, to account for LED wavelength-dependent attenuation, further parametric optimization simulations were performed for each LED to determine optimal component positions using a range of lenses (Fig. S1 in the Supplemental Material) and subsequently performing a detailed series of simulations using the 31.8-mm FL lens (Fig. S2 in the Supplemental Material). Flux percentages were plotted with their respective parametric locations, and final component parameters were acquired to determine final prototype placement for each LED and lens component (Table 6). Furthermore, results determined that a smaller spot size distribution led to higher percent transmission due to the ability to absorb more of the LED’s reflected rays. In addition, it was evident that, although there is an optimal parametric location for each component to achieve high percent transmission, the transmission percentage relied more on the LED-to-lens spacing than the lens-to-mirror or LED-to-mirror. For example, peak percent transmission for the 365-nm LED was found to be 6.80% at the following $(x_{\mathrm{LED}},x_{\text{lens}})\;\mathrm{mm}$ location configurations: (80, 32) mm and (81, 34) mm, 6.79% at the following $(x_{\mathrm{LED}},x_{\text{lens}})\;\mathrm{mm}$ location configurations: (80, 32) mm and (81, 33) mm, and 6.77% at the following $(x_{\mathrm{LED}},x_{\text{lens}})\;\mathrm{mm}$ location configurations: (79, 30) mm, (79, 33) mm, (80, 30) mm, (82, 34) mm, and (82, 35) mm. Hence, it was concluded that a minimum distance of 46 mm and a max distance of 50 mm between the LED and lens will provide a high transmission range of 6.77% to 6.80%. Results of the LED-to-lens distance influence on the final transmission percentage were consistent for all sensitivity results.

**Table 6:** Final component locations based on parametric sensitivity results for each LED using the 31.8-mm FL lens.

Wavelength (λ)	LED x axis location (mm)	Lens x axis location (mm)	Highest transmission (%)
365	80	32	6.80
375	81	33	7.02
395	81	32	6.52
405	80	31	6.53
420	83	35	6.06
450	82	33	4.82
470	82	33	4.91
490	93	33	6.81
515	82	32	5.84
520	83	34	4.66
525	84	37	5.95

### 3.2 Benchtop Testing Results and Discussion

#### 3.2.1 LED spectroradiometric testing results and discussion

**Fig. 6:**
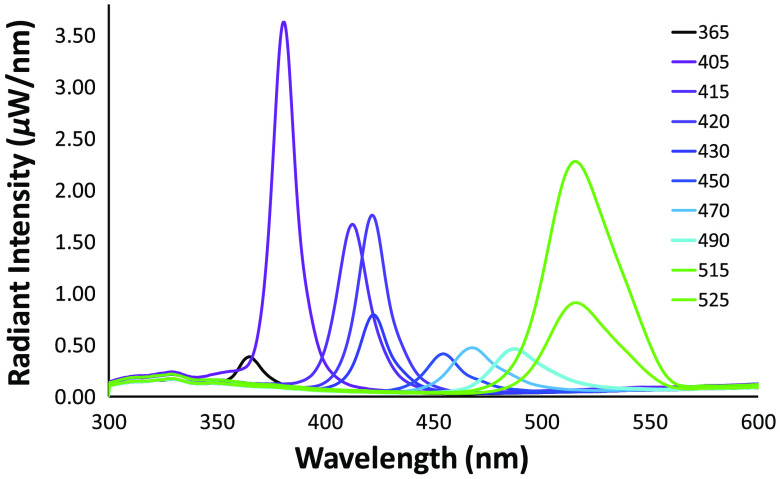
Spectroradiometric optical power output as measured at the microscope stage for each LED illumination band when measured at the end of the light path with LEDs set at maximum intensity. Measurements were made by placing an integrating sphere on the stage aligned directly over the objective (end of light path) and measuring the total spectral irradiance.

Spectroradiometric testing was performed to assess the illumination intensity available at the microscope stage for each LED (Fig. 6). In comparing theoretical model results with stage measurements, it was clear that there were light losses between the entrance aperture of the LLG and the stage of the microscope. There are light losses present throughout the system. The first stage in which the system experiences light losses is when the rays are collimated through the lens. Next, there are light losses present when rays are reflected by the mirror onto the LLG entrance aperture and finally through the LLG and the microscope system (objective and stage). Due to the serious light losses present in our system, image acquisition times were determined to fill the dynamic range of the detector for the most intense (brightest) wavelength band.

### 3.3 Feasibility Testing Results and Discussion

#### 3.3.1 Spectral library

**Fig. 7:**
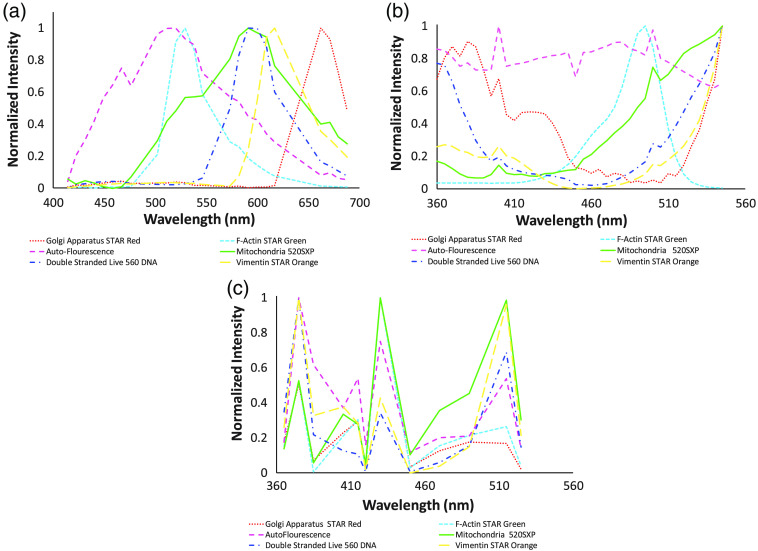
Spectral libraries were compiled for three spectral microscopy systems: (a) a commercial emission scanning spectral confocal microscope (Zeiss LSM 980), (b) an excitation-scanning system using TFTFs for spectral tuning and implemented on an inverted widefield microscope platform (TFTF-based HIFEX), and (c) an excitation-scanning system using LEDs for spectral excitation and implemented on an inverted widefield microscope platform (LED-based HIFEX).

To build each spectral library, spectral images from each single label control sample were acquired using the HIFEX and LED-based HIFEX systems and corrected to a flat spectral response. Spectral images were also acquired from the Zeiss LSM 980 commercial emission-scanning spectral confocal microscope system. For each image of each single-label control sample, ROIs were selected, and the mean spectrum of each ROI was measured. To achieve a strong SNR for each label in the spectral libraries, high intensity ROIs (data not shown) were used for spectra selection. A separate spectral library was created for each spectral microscope system: LSM 980, TFTF-based HIFEX, and LED-based HIFEX (Fig. 7). Spectral libraries were plotted in Excel for visualization and subsequently imported into MATLAB for linear unmixing. Spectral library results for the LSM 980 microscope system (Fig. 7) were in agreement with manufacturer (Abberior GmbH) label emission spectral properties available on their website for emission spectrums of STAR Red, STAR Green, 520SXP, Live 560, and STAR Orange. Note that several laser lines (Table 5) were used for excitation, so we were able to satisfactorily excite each of the labels. Moreover, some wavelength bands were discarded to remove the notch created in the emission profile where it overlaps the excitation profiles of the laser due to a mechanical finger that is placed in front of the specific spectral detector to not damage it from the reflected laser light. As expected, the AF profile is very broad, likely indicating AF contributions from multiple molecules, such as collagen and possibly FAD.

The HIFEX system spectral library produced spectra that are similar to those provided in manufacturer data sheets. The excitation wavelength for STAR red approximately begins around 525 nm with a max peak at 638 nm, and the emission approximately ranges between 650 and 800 nm with a peak at 655 nm. Thus, we saw a notable increase in that label’s spectral signature beginning at the 530 nm mark. Because the dichroic cutoff for HIFEX systems is 555 nm, we cannot excite the sample at any wavelength above this cutoff. Spectral signature peaks past the 430 nm for the HIFEX system [Fig. 7(b)] corresponded with the manufacturer data for the rest of the labels in the spectral library. It can be seen in the spectral library results [Fig. 7(b)] that there are high background noise and a broad spectrum for autofluorescence signal. Labels with excitation peaks outside the range can be seen to be increasing near the end of the scan range, but there are also UV components (secondary peak excitation wavelengths) that may be used to spectrally separate the contribution from each label—an advantage of using spectral imaging. With spectral imaging, along with finding a peak value, the overall shape of the wavelengths is different. These shape differences present in the spectral signature made it possible to separate contributions from the different florescent labels even though the peak excitation wavelength for every fluorescent label was not contained within the scan range.

For the LED-based HIFEX spectral library [Fig. 7(c)], label peaks were also present at expected wavelengths; however, high cellular autofluorescence and background noise signals were mixed into the overall signal of each label. In addition, it is worth noting that the wavelength sampling in the LED-based HIFEX system was reduced compared with the TFTF-based HIFEX. Thus, the library for the LED-based HIFEX does not appear to be as continuous. Furthermore, some wavelengths provided much lower spectral output than others, and although this has been corrected for by measuring the spectral response of the system, it is still likely that the SNR at those lower wavelengths is lower than the SNR at the brighter illumination wavelengths. Therefore, the measurements at the lower illumination wavelengths are likely less reliable. Although not encountered in this study, it is also worth noting that the use of a maximal illumination output with a fixed acquisition time for each spectral band could lead to image saturation for specific spectral bands in which a high signal is present. To mitigate these effects, it is possible to vary or reduce the excitation intensity of specific wavelengths using the LED-based HIFEX system. Despite the lower number of wavelengths sampled, we were still able to identify signals from the fluorescent labels, as shown in Fig. 7.

#### 3.3.2 Image analysis results and discussion

**Fig. 8:**
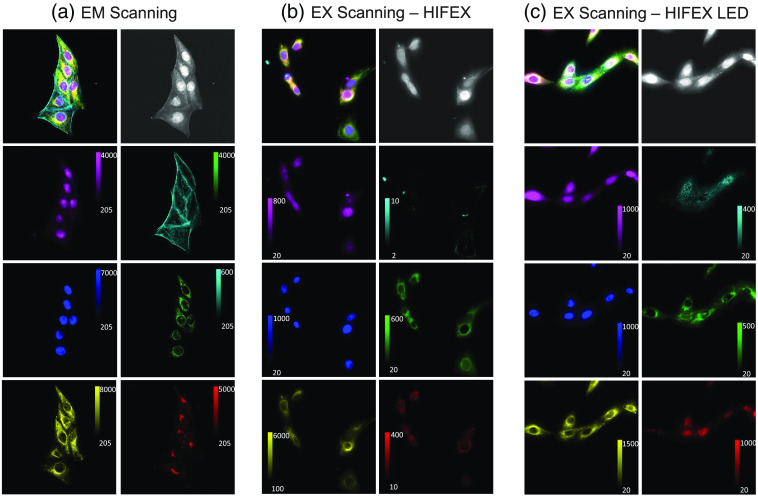
Comparison of image data acquired from three spectral imaging microscopy systems: (a) a commercial emission-scanning spectral confocal microscope (Zeiss LSM 980), (b) HIFEX: a previously reported excitation-scanning spectral widefield microscope that uses a TFTF for spectral tuning, and (c) an LED-based excitation-scanning spectral widefield system that uses an array of LEDs and multifurcated mirror for spectral illumination. For each image panel, the upper left image displays the results of linear unmixing as a merged and false-colored composite image, and the upper right image displays the sum of all wavelength bands for the spectral image stack (total fluorescence). Individual components identified through linear unmixing were visualized using a color lookup table to match the colors shown in the false-colored and merged image and are described from left-to-right and top-to-bottom: magenta, cellular autofluorescence; cyan, F-actin labeled with Star Green; blue, double-stranded DNA labeled with Live 560; green, mitochondria labeled with STAR 520SXP; yellow, vimentin labeled with STAR Orange; red, Golgi apparatus labeled with STAR Red.

**Fig. 9:**
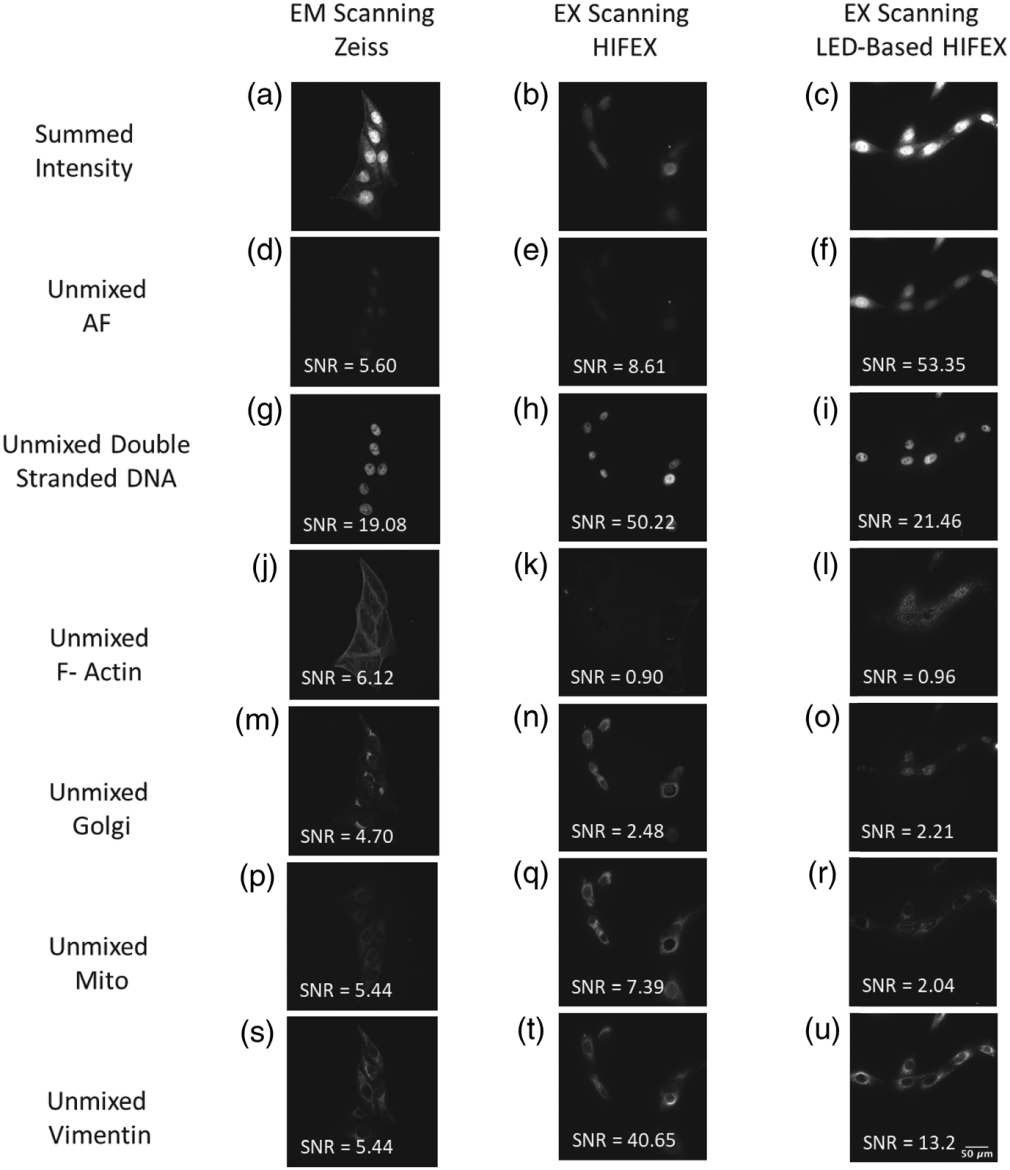
SNR comparison of image data acquired from three spectral imaging microscopy systems: a commercial emission-scanning spectral confocal microscope (Zeiss LSM 980), a previously reported excitation-scanning spectral widefield microscope that uses a thin-TFTF for spectral tuning, and the excitation-scanning spectral widefield system that uses an array of LEDs and multifurcated mirror for spectral illumination. (a)–(c) Summed fluorescence intensity was used to visualize raw image data from each system. (d)–(u) SNR for each label was calculated for each system.

For visualization purposes, an image of the total fluorescence was computed by summing the intensity at each wavelength for each of the three systems [Figs. 8(a)–8(c), top right]. Image stacks containing AF, STAR Red, STAR Green, 520SXP, Live 560, and STAR Orange were linearly unmixed using non-negatively constrained linear spectral unmixing, which calculates the abundance of each label contained in the spectral library for each pixel of the spectral image stack and in turn produces the unmixed abundance image for each label [Figs. 8(a)–8(c)]. Linearly unmixed images were merged and false colored for all three systems for visualization purposes [Figs. 8(a)–8(c), top left]. Autofluorescence was best discriminated by the LED-based HIFEX system, which displayed a sixfold increase over the excitation scanning-based HIFEX and over a ninefold increase over the Zeiss emission scanning confocal microscope; this was evident from the SNR results [Figs. 9(d)–9(f)]. Double-stranded DNA [Figs. 9(g)–9(i)] was best discriminated by the HIFEX system (SNR = 50.22) followed by the LED-based HIFEX (SNR = 21.46) and then the Zeiss system (SNR = 19.08). F-actin was more clearly delineated in the Zeiss system [Figs. 9(j)–9(l)], which is primarily due to the optical sectioning capabilities provided by the Zeiss LSM 980. Clear delineation of F-actin is a result of confocal imaging and the ability to image an optical slice of the small fibular structure and remove the out of focus light. In contrast, widefield imaging does not allow for an optical section, and thus there were likely some structures lost. In future studies, it is possible that a higher labeling density of F-actin may result in improved detection through both excitation scanning systems. Golgi SNR was low across all three systems [Figs. 9(m)–9(o)] but highest in the Zeiss system (SNR = 4.7), followed by the HIFEX (SNR = 2.48) and LED-based HIFEX (SNR = 2.21) systems. Because both the LED-based HIFEX and HIFEX systems implemented a diachronic cutoff at 555 nm, SNRs for individual unmixed labels with excitation peaks past the cutoff were expected to be low. Mitochondria SNR was highest in the HIFEX system (SNR = 7.39), followed by the Zeiss and LED-based systems, respectively [Figs. 9(p)–9(r)]. Vimentin was most delineated through the HIFEX system with an SNR of 40.65 followed by LED-based HIFEX (SNR = 13.2) and Zeiss (SNR = 5.44).

## 4 Conclusion and Future Work

HSI technology has undergone continuous development from its beginning in remote sensing to the wide range of applications used today. In the fluorescence microscopy field, HSI has shown great utility for enabling specific and quantitative measurement of fluorescent labels and autofluorescence. In this paper, we introduced an innovative approach—rapid-HSI, as a needed improvement and advancement in existing HSI techniques. Our approach shows potential for improving the speed at which spectral microscopy measurements may be using LEDs for spectral excitation, allowing for rapid wavelength-switching times. The long-term goal of this approach is to develop a system that will allow the user to obtain simultaneous measurements of multiple cell-signaling molecules, cellular anatomical structures, and indicators of cell physiology. Here, we have reported on the initial design of a LED-based spectral excitation-scanning spectral microscope that uses a multifaceted mirror to combine the optical output from many LEDs. Benchtop testing and initial feasibility studies demonstrate that this approach is capable of identifying signals from many fluorescent labels in a multilabel sample. Wavelength switching times for this system are very short (10 to 20  μs); however, image acquisition times were longer than anticipated due to optical transmission losses in the prototype, compared with the ray trace model, and in the microscope body itself. Future work will focus on increasing the illumination power by mitigating transmission losses within the system as a whole and by increasing the power provided by individual wavelength-specific LEDs through pulsing, improved heat dissipation, and selection of higher output LED models. Increased spectral excitation power will, in turn, allow for increased SNR imaging and/or decreased spectral image acquisition times, both of which are necessary for live-cell dynamic imaging.

## Code, Data, and Materials Availability

Image data sets and example MATLAB image analysis code are available through the University of South Alabama BioImaging and BioSystems website at https://www.southalabama.edu/centers/bioimaging/resources.html.
