# Supplementary material for: Multifaceted mirror array illuminator for fluorescence excitation-scanning spectral imaging microscopy
Source: J Biomed Opt. 2023 Feb 7;28(2):026502. doi: 10.1117/1.JBO.28.2.026502 (PMC9907356; doi:10.1117/1.JBO.28.2.026502)
Supplement: Supplementary file 1 [file JBO_028_026502_SD001.pdf]

Supplementary Material

Multifaceted Mirror Array Illuminator for Fluorescence Excitation-Scanning Spectral Imaging Microscopy

Marina Parker, Samuel A. Mayes, Craig M. Browning, Joshua Deal, Samantha Gunn-Mayes, Naga S. Annamdevula, Thomas C. Rich, Silas J. Leavesley

Journal of Biomedical Optics

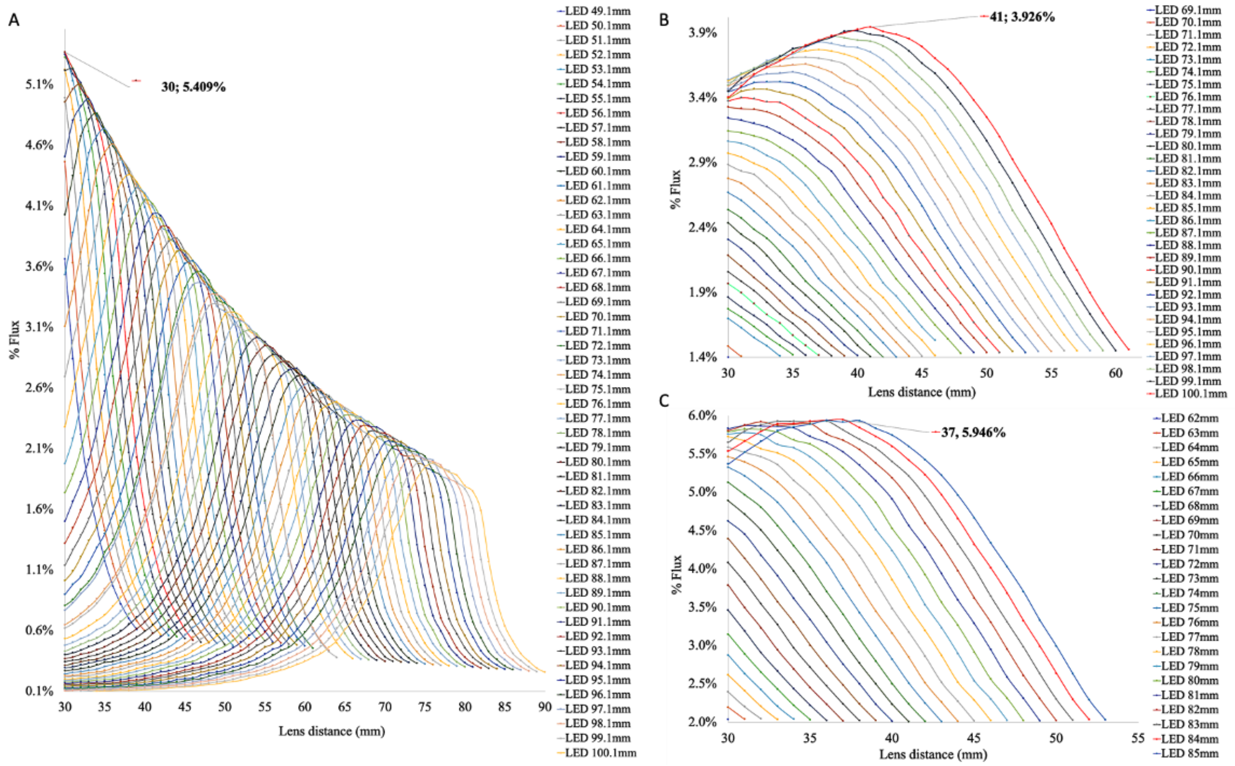

Fig. S1 *Parametric sensitivity results for the three lenses (A) 19.1 mm FL, (B) 38.1 mm FL, and (C) 31.8mm FL modeled in TracePro using the 525 nm LED. Each study evaluated a range of possible positions for both LED and lenses. LED positions were evaluated for a range of* x_{LED} *= [49.1 – 100.1] (A),* x_{LED} *= [69.1 – 100.1] (B), and* x_{LED} *= [62 – 85] (C) while lens ranges were* x_{lens} *= [30.1 – 90] (A),* x_{lens} *= [30 – 61] (B), and* x_{lens} *= [30 – 53] (C)]. The annotation indicates the lens distance that provided the peak percent transmission, and the percent transmission. The peak percent transmission values were found to be 5.41% for the 19.1 mm FL lens (A), 3.93% for the 38.1 mm FL lens (B), and 5.95% for the 31.8 mm FL lens (C).*

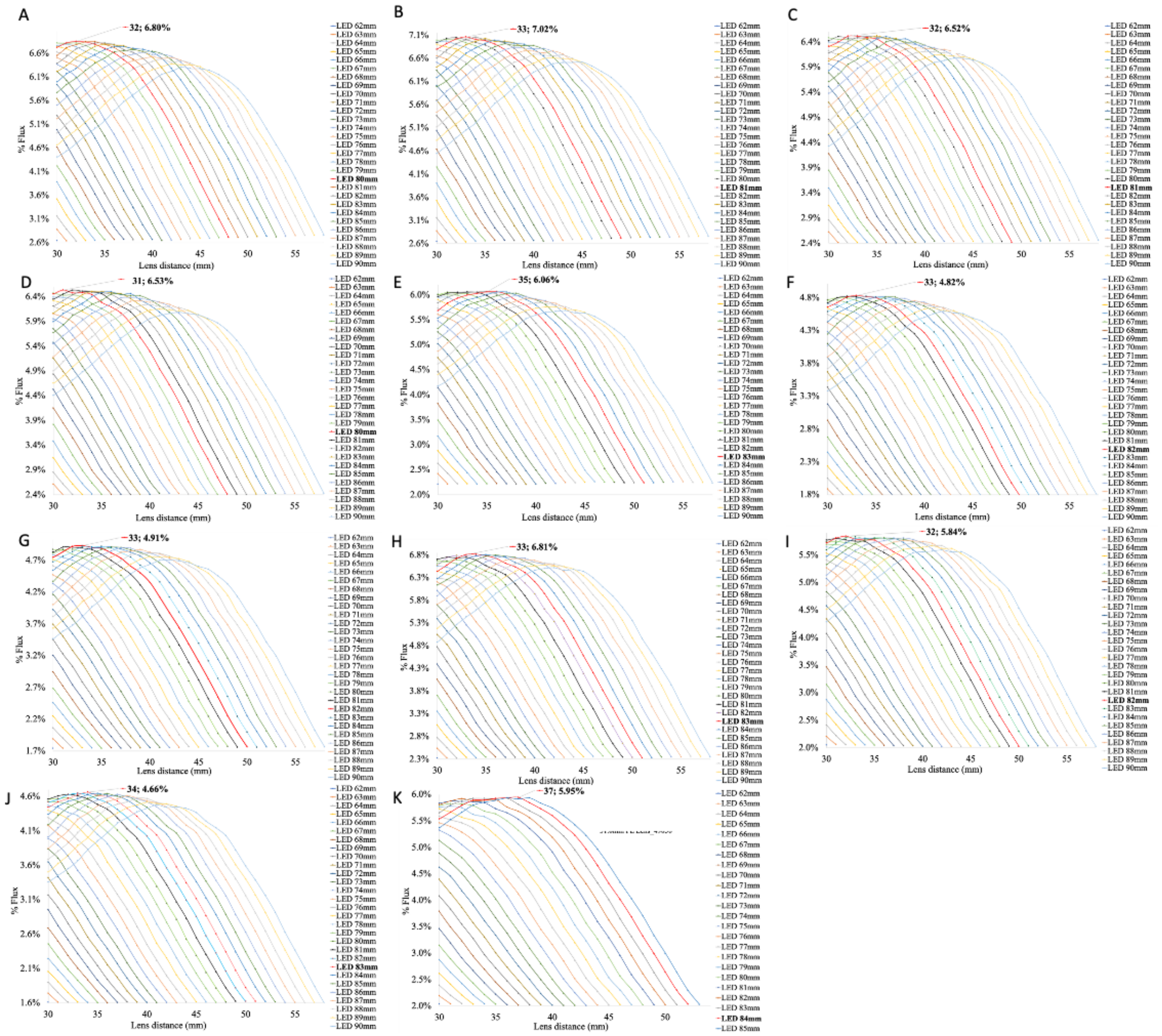

Fig. S2 *Parametric sensitivity results for each LED using the 31.8 mm FL lens for the following LEDs: (A)365, (B) 375, (C) 395, (D) 405, (E) 420, (F) 450, (G) 470, (H) 490, (I) 515, (J) 520, and (K) 525. Peak transmission values for LED wavelength are summarized in Table 6.*
